# Liraglutide and Dulaglutide therapy in addition to SGLT-2 inhibitor and metformin treatment in Indian type 2 diabetics: a real world retrospective observational study

**DOI:** 10.1186/s40842-018-0061-8

**Published:** 2018-05-09

**Authors:** S. Ghosal, B. Sinha

**Affiliations:** 1 Nightingale Hospital, 11 Shakespeare Sarani, Kolkata, India; 2 AMRI Hospitals, JC-16-17, Salt Lake City, Kolkata, 700091 India; 3 Kolkata, India

**Keywords:** Diabetes, Liraglutide, Dulaglutide, SGLT-2i, HbA1c, Weight

## Abstract

**Background:**

Therapy for Type 2 diabetes (T2D) has been transformed by the introduction of newer agents like Glucagon like Peptide Receptor Agonists (GLP-1RA) and Sodium-glucose linked transporter inhibitors (SGLT2i). However with co-initiation of SGLT2i and GLP-1RA in the DURATION 8 trial an improvement in HbA1c was noted but the beneficial effect was not equal to the sum of its parts. In view of this we proceeded to test the hypothesis that sequential addition of GLP-1RA therapy to metformin and SGLT-2i may be more beneficial.

**Methods:**

A retrospective real world observational case note study conducted in two diabetes care centres in India analyzed the first 60 consecutive T2D patients who could afford this therapy and had not achieved their glycaemic target (HbA1c < 7%)on metformin and SGLT2i. All these patients were additionally treated with either Dulaglutide or Liraglutide and followed up for 13 weeks.

**Results:**

Across the entire 13-week study period, both liraglutide and dulaglutide proved to be an excellent add on to metformin and SGLT-2 inhibitor. There was significant reduction in HbA1c and body weight. Liraglutide had an additional significant impact on systolic blood pressure reduction in contrast to the dulaglutide arm. Comparatively, liraglutide and dulaglutide achieved similar metabolic control. However, a larger proportion of patients achieved HbA1c below 7.0% in the liraglutide arm (63.3%) compared to the dulaglutide arm (30%) and this difference was statistically significant.

**Conclusion:**

In this retrospective study in Indian type 2 diabetic patients poorly controlled with metformin and SGLT-2 inhibitor we found a meaningful impact of adding a GLP-1 RA on all metabolic parameters. There were additional advantages seen with liraglutide as far achieving target HbA1c of less than 7% and also on the quantum of weight loss and systolic blood pressure reduction.

## Background

The combination of Glucagon like Peptide Receptor Agonists (GLP-1RA) with a Sodium-glucose linked transporter inhibitor (SGLT2i) addresses many of the pathophysiological defects seen in Type 2 Diabetes (T2D), according to certain researchers [1]. In the EDICT trial, using the strategy of treating T2D, using multiple agents addressing the pathophysiological defects of the disease (insulin resistance, beta-cell dysfunction and hyperglucagonaemia) was found to be superior to the traditional step wise approach to glycaemic control in terms of HbA1c reduction and reduction of hypoglycaemia [2]. There have also been robust positive outcomes from the recent cardiovascular outcome trials (CVOT) with SGLT2i (empagliflozin and canagliflozin) and GLP-1 RA(liraglutide and semaglutide), establishing cardiovascular benefits attributable to these agents [3–6]. Hence there is a good scientific rationale for using them in combination.

The recent DURATION 8 study, testing the above rationale, demonstrated an additive effect on weight and systolic blood pressure reduction but not HbA1c reduction when exenatide LAR, a GLP-1 RA and dapagliflozin, a SGLT2i were used in combination as a co-initiation strategy [7, 8]. However multiple observational studies suggested that GLP-1 RA and SGLT2i are additive from a metabolic perspective as well, when SGLT2i was added to GLP-1 RA (sequential initiation instead of co-initiation) [9, 10]. This is contrary to a real world scenario where usually injectable are added only when oral drugs fail. This differing data therefore poses a conundrum for the physician as to whether to combine these drugs together or not, particularly keeping in mind that these drugs are very expensive. In a resource poor setting such as India where patients pay ‘out-of-pocket’, injectable therapies are usually used as a third-line agent when oral therapy fails. Hence, data on this combination mimicking a real-life setting i.e. oral therapy followed by injectable would be useful in guiding physician choices for intensification options in Type 2 diabetes.

We therefore designed this study to look into the sequential additive benefit of GLP-1 RA therapy to preexisting SGLT2i and Metformin therapy as well as comparing Dulaglutide with Liraglutide, in combination with SGLT2i and Metformin. The aim of this study is to evaluate real-life data from clinical practice using this combination of drugs and assess how the results compare with the available data from randomized controlled trials.

## Methods

A retrospective, real world observational study to evaluate the efficacy of triple-anti-hyperglycemic agent therapy namely metformin, sodium glucose co-transporter 2 Inhibitors (SGLT2i) and glucagon like peptide receptor agonists (GLP-1 RAs) for patients failing on a combination of full dose metformin 2000 mg/day and SGLT2ifor at least 3 months, was conducted in the outpatient clinics associated with two hospitals in Kolkata, India, from May 2016 to August 2016. The baseline characteristics of the patients included for analysis are detailed in Table 1.

**Table 1:** Baseline Characteristics of the Patients (*N* = 60)

Demographic profile:		Liraglutide, *N* = 30	Dulaglutide, *N* = 30	*P* value
Male, *n* (%)	28(46.7%)	13 (43.3%)	15 (50%)	0.605
Female, *n* (%)	32 (53.3%)	17 (56.7%)	15 (50%)	
Age(years), Mean ± SEM	47.75 ± 1.22	47.33 ± 1.91	48.17 ± 1.55	0.736
Height (centimeters), Mean ± SEM	161.53 ± 1.2	159.97 ± 1.52	163.10 ± 1.83	0.193
Body weight(Kg), Mean ± SEM	88.3 ± 1.68	89.43 ± 2.60	87.17 ± 2.16	0.506
Diabetes duration, (years), Mean ± SEM	6.07 ± 0.70	5.55 ± 0.94	6.59 ± 1.03	0.46
SBP(mmHg), Mean ± SEM	136.07 ± 1.84	137.83 ± 2.50	134.30 ± 2.71	0.342
DBP(mmHg), Mean ± SEM	82.1 ± 1.15	83.47 ± 1.58	80.73 ± 1.64	0.236
BMI(kg/m2), Mean ± SEM	34.99 ± 5.66	34.92 ± 0.86	32.84 ± 0.77	0.078
BMI – 25 -29.9	10 (16.67%)			
BMI - 30-34.9	30 (50%)			
BMI - 35-39.9	12 (20%)			
BMI - ≥40	8 (13.33%)			
FPG(mg/dL), Mean ± SEM	167.83 ± 7.04	160.13 ± 8.17	175.53 ± 11.45	0.278
HbA1c(%), Mean ± SEM	8.46 ± 0.17	8.49 ± 0.26	8.43 ± 0.21	0.847
On Metformin, *n* (%)	60 (100%)	30 (100%)	30 (100%)	1.00
On SGLT-2is, *n* (%)	60 (100%)	30 (100%)	30 (100%)	1.00

After clearance from the local ethics committee (Nightingale Hospital ethics committee), the case notes of the first 30 consecutive patients who had been commenced on Metformin plus SGLT2i plus Dulaglutide, in addition to the first 30 patients who had been commenced on Metformin plus SGLT2i plus Liraglutide were collated after signing patient consent form to use their data for publication purpose. The ethics committee decided that consent of the patients was not required as this was a completely retrospective study of case notes with no intervention required.

Furthermore when the patients’ data was entered into the database the patient could only be identified by a number; so there was no chance of the patients’ confidentiality being compromised.

After adequate counseling, patients *who could* ***afford*** *this expensive combination of medications for at least 3 months* were commenced on this therapy. The following inclusion and exclusion criteria decided by the two centres:

### Inclusion criteria

- Adult Type 2 diabetics with HbA1C ≥ 7.0% on Metformin plus SGLT2i
- Body mass index (BMI) ≥ 25 kg/m^2^,
- eGFR> 45 ml/min

### Exclusion criteria

- Type 1 Diabetes
- Pregnancy
- Deranged liver function tests
- Any major organ system disease as determined by physical examination, medical history and screening blood tests
- Recent insulin therapy
- Recent anti obesity therapy
- Recent treatment with any other oral anti diabetics
- History of pancreatitis
- Family history of Medullary Thyroid cancer or MEN 2

Patients received treatment as per routine standard of care. All anti hypertensives, anti hyperlipidaemics and anti platelet agents and other preexisting medications (not related to diabetes) were continued as per the patients’ requirements. All patients’ records with respect to age, gender, height, body weight, body mass index (BMI), duration of diabetes, glycosylated hemoglobin (HbA1c), fasting plasma glucose, blood pressure and adverse effects were collected from the case note database. Blood glucose was measured by hexokinase method and HbA1c was measured by high performance liquid chromatographic (HPLC) method (Bio-RAD D-10, Bio-RAD, Hercules, CA, USA).

All 30 patients had been maintained on Liraglutide in a dose of 1.2 mg per day and all 30 patients on dulaglutide 1.5 mg dose once-weekly during the 13 weeks of the study period. Both the arms were well matched as far as baseline characteristics were concerned. (Table 1).

All 60 patients received either of the SGLT-2 Inhibitors namely dapagliflozin 10 mg/day (*n* = 28), canagliflozin 100 mg/day (*n* = 20), empagliflozin 10 mg/day (*n* = 12), as per the treating physicians’ decision. All patients were on Metformin 2000 mg per day. Other OADs had not been initiated.

### Statistical methods

Descriptive statistical analysis were carried out with SAS (Statistical Analysis System) version 9.2 for windows, SAS Institute Inc. Cary, NC, USA and Statistical Package for Social Sciences (SPSS Complex Samples) Version 21.0 for windows, SPSS, Inc., Chicago, IL, USA, with Microsoft Word and Excel being used to generate graphs and tables. Results on continuous measurements are presented as Mean ± SEM and results on categorical measurements are presented in Number (%). Significance is assessed at a level of 5%.

The following assumptions were made of the data: 1) Cases of the samples should be independent, 2) The populations from which the samples are drawn have the same variance (or standard deviation) and 3) The samples are drawn from different populations are random.

Normality of data was tested by Anderson Darling test, Shapiro-Wilk, Kolmogorov-Smirnoff test and visually by QQ plot. Paired t-test was used to find the significance of study parameters within groups of patients measured on two occasions. Chi-square/ Fisher Exact test was used to find the significance of study parameters on categorical scale between two or more groups.

## Results

### Impact of adding either Dulaglutide or Liraglutide to metformin and SGLT2 inhibitors

#### Dulaglutide

Adding dulaglutide to the combination of metformin 2000 mg/day and aSGLT2i resulted in a significant reduction in fasting plasma glucose (− 41.87 ± 12.72 mg/dL; *p* = 0.003) and HbA1c at 3 months follow up (− 1.017 ± 0.22%; *p* < < 0.001) [Table 2]. The reduction in glycemic parameters was accompanied by a significant impact on body weight (− 4.20 ± 0.47 kg; *p* < < 0.001 and BMI (− 1.53 ± 0.21%; *p* < 0.001) without an impact on blood pressure.

**Table 2:** Change in study parameters during the follow-up period

Cohort	Dulaglutide, *n* = 30				Liraglutide, *n* = 30			
Parameter	Baseline Mean ± SEM	Follow-up Mean ± SEM	Change Mean ± SEM	*P*	Baseline Mean ± SEM	Follow-up Mean ± SEM	Change Mean ± SEM	*P*
Body weight (kg)	87.17 ± 2.16	82.97 ± 2.05	−4.20 ± 0.47	< 0.001	89.43 ± 2.60	83.60 ± 2.35	−5.83 ± 0.87	< 0.001
BMI (kg/m^2^)	32.84 ± 0.77	31.312 ± 0 .68	−1.53 ± 0.21	< 0.001	34.92 ± 0.868	32.650 ± 0.77	−2.27 ± 0.33	< 0.001
SBP (mmHg)	134.30 ± 2.71	130.87 ± 2.49	−3.43 ± 2.97	0.258	137.83 ± 2.503	127.60 ± 2.07	−10.23 ± 2.36	< 0.001
DBP (mmHg)	80.73 ± 1.64	78.43 ± 0.914	− 2.30 ± 1.79	0.208	83.47 ± 1.587	80.67 ± 1.51	− 2.80 ± 1.85	0.141
FPG(mg/dl)	175.53 ± 11.45	133.67 ± 6.90	− 41.87 ± 12.72	0.003	160.13 ± 8.17	115.93 ± 5.69	− 44.20 ± 8.05	< 0.001
HbA1c (%)	8.43 ± 0.21	7.411 ± 0.15	−1.017 ± 0.22	< 0.001	8.49 ± 0.26	6.95 ± 0.21	− 1.547 ± 0.22	< 0.001

#### Liraglutide

As with dulaglutide, treatment with liraglutide in addition to Metformin and a SGLT2i resulted in a significant reduction in fasting plasma glucose (− 44.20 ± 8.05 mg/dL; *p* < 0.001), HbA1c (1.547 ± 0.22%; *p* < 0.001), weight (− 5.83 ± 0.87 kg; *p* < 0.001) and BMI (2.27 ± 0.33&; *p* < 0.001) at 3 months follow up [Table 2]. However in contrast to dulaglutide, liraglutide was associated with a significant reduction in systolic blood pressure (− 10.23 ± 2.36 mm of Hg; *p* < 0.001) [Table 2].

### Dulaglutide versus Liraglutide failing OHA regime

We analyzed the data to compare the effects of dulaglutide with liraglutide, when they were both added to existing therapy of Metformin and SGLT2i.

#### Glycemic parameters

There was a comparable reduction in FPG and HbA1c in both the arms. However, 63.3% of patients in the liraglutide achieved an HbA1c of < 7.0% compared to only 30% in the dulaglutide arm. This difference was statistically significant (0.019) [Table 3].

**Table 3:** Proportion of patients achieving HbA1c less than 7%

			Follow-up A1c < 7%		Total	*p* (2-sided)
			No	Yes		
Cohort	Dulaglutide	Number of Patients	21	9	30	0.019
		%	70.0%	30.0%	100.0%	
	Liraglutide	Number of Patients	11	19	30	
		%	36.7%	63.3%	100.0%	
Total		Number of Patients	32	28	60	
		%	53.3%	46.7%	100.0%	

#### Weight

**Fig. 1:**
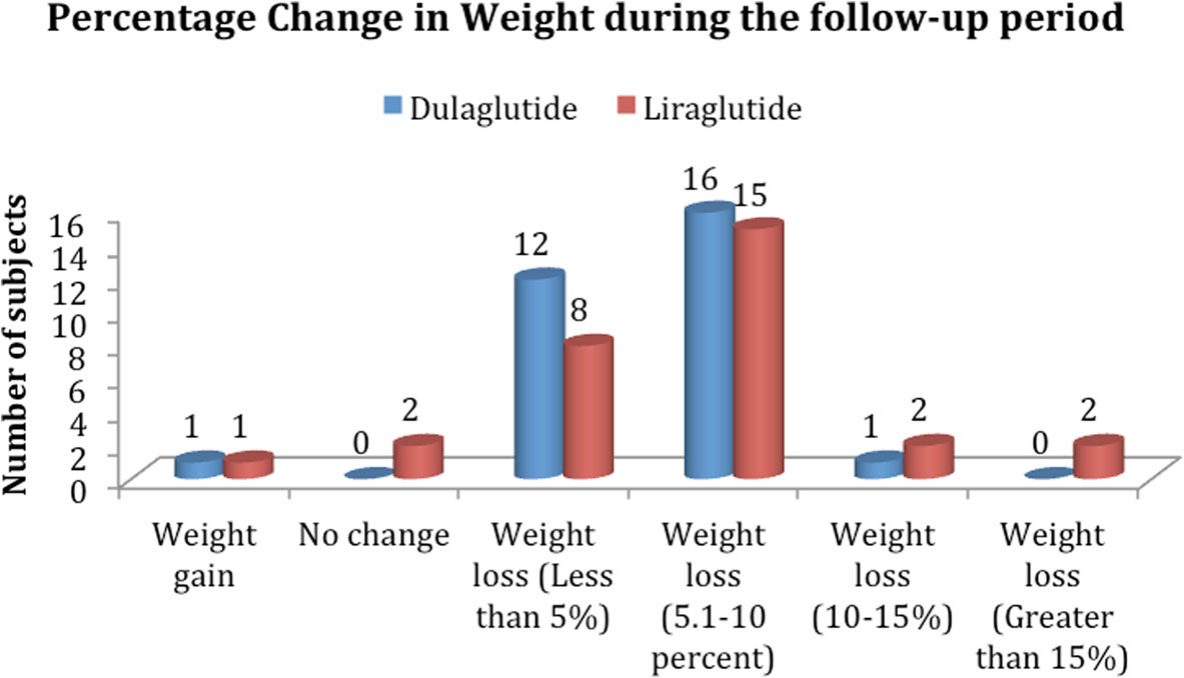
Percentage Change in Weight during the follow-up period

There was comparable weight loss in both the arms [Table 4]. A similar proportion of patients in both the arms experienced a weight loss between 5 and 10% (53.3% with dulaglutide vs. 50.0% with liraglutide). A highly significant weight loss of greater than 15% from baseline was seen in 6.7% patients in the liraglutide arm, but not in the dulaglutide arm [Fig. 1].

**Table 4:** Percentage Reduction in Study Variables

Study Variables	Liraglutide, *N* = 30	Dulaglutide, *N* = 30	*p*
Percent Change in Body weight Mean ± SEM	−5.83 ± 0.87	−4.2 ± 0.46	0.103
Percent Change in BMI, Mean ± SEM	−2.27 ± 0.33	1.53 ± 0.20	0.061
Percent Change in SBP, Mean ± SEM	−10.23 ± 2.36	−3.43 ± 2.98	0.079
Percent Change in DBP, Mean ± SEM	−2.8 ± 1.85	−2.3 ± 1.79	0.847
Percent Change in FPG, Mean ± SEM	−44.2 ± 8.05	−41.87 ± 12.72	0.877
Percent Change in HbA1c, Mean ± SEM	− 1.55 ± 0.22	−1.02 ± 0.22	0.091

#### Blood pressure

There was similar reduction in systolic and diastolic blood pressure in both the arms [Table 4].

### Dulaglutide vs. Liraglutide: Impact on the metabolic composite of HbA1creduction AND weight loss

**Fig. 2:**
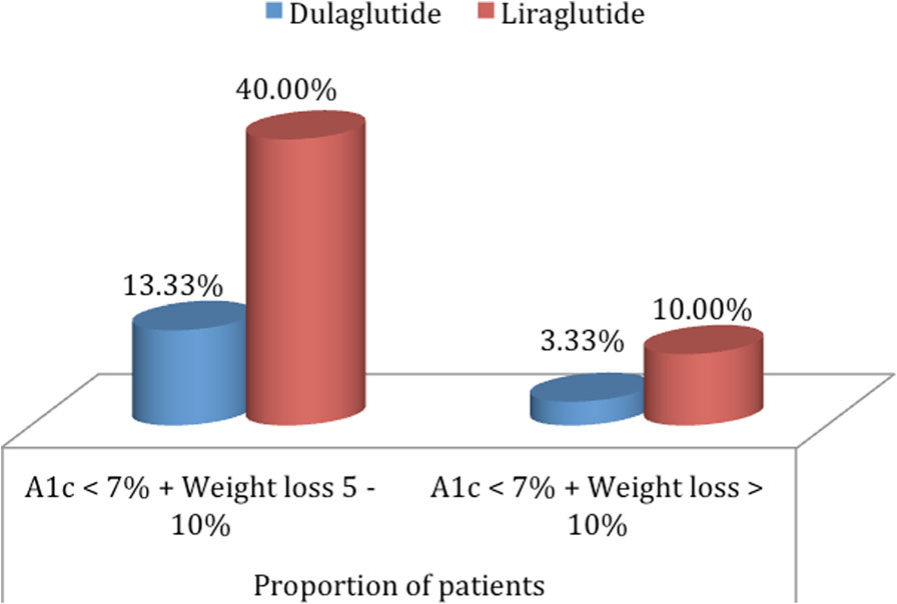
Proportion of patients achieving the HBA1C & weight composite

The composite end point of HbA1creduction to below 7% and a greater than 5% of body weight loss was attained by 5 (16.7%) patients in the dulaglutide arm, while 20 (50%) patients on liraglutide arm reached this composite (*p* = 0.75). (Fig. 2).

### Adverse events

No serious adverse event was reported during this 3 month follow up. There was no hypoglycaemia reported. The commonest adverse event was nausea in 14 (23%) patients, 6 (20%) in the dulaglutide group compared to 8 (26.6%) in the liraglutide group. One patient who was on liraglutide complained of 1 -2 episode of vomiting and diarrhoea, which responded to a week’s treatment with a proton pump inhibitor and domperidone. There were no complaints of diarrhoea, vomiting or abdominal pain from the patients on dulaglutide. Two patients complained of genital irritation, which responded to topical anti mycotics. Both these patients were on dapagliflozin, with one of them taking dulaglutide and the other being on liraglutide. There were no dropouts from the cohort over the 3-month period of our study, which though surprising in the real world, was quite opportune.

## Discussion

Recent publications of positive CV outcome trials with SGLT2i (empagliflozin and canagliflozin) and GLP1 RA (liraglutide and semaglutide) and the positive effects of treatment with these drugs utilizing the pathophysiological defects of T2D have caused a paradigm shift in the way the disease is treated [1–4]. Quite naturally, researchers have been interested to study the effects of co-initiating SGLT2i and GLP-1 RA. The DURATION 8 study was published recently showing a robust weight loss and reduction in systolic blood pressure in the dapagliflozin plus Exenatide arm [7]. Strangely, this benefit did not extend to HbA1c reduction [8]. Nauck et al. in their editorial accompanying the publication of DURATION 8 postulated that since the additive effect of the two molecules were “disappointing” when co initiated, it would be prudent to test their effectiveness when started sequentially as would happen “probably in most clinical cases” [8].

A real-world observational data looked at the effect of sequential addition of canagliflozin after receiving GLP-1RA for 30 months [9]. This study documented a very modest HbA1c reduction. In another small retrospective study (*n* = 14) from the United Kingdom sequential addition of GLP-1RA followed by SGLT-2 inhibitor was analyzed [10]. On addition of a GLP1 RA, HbA1c came down by 8 mmol/mol (0.7%), with an associated 4.9 kg weight loss. After 20 weeks, a SGLT-2 inhibitor was added in sequence and followed up for 48 weeks. There was an additional significant reduction in HbA1calong with 5.47 kg weight loss.

However, the authors find in their clinical practice, injectables are tried or accepted by patients only after oral therapy has been exhausted, for obvious reasons. In the studies described above, SGLT2i (oral therapy) was added to preexisting injectable (GLP-1 RA) therapy or the drugs were co initiated, differing from a real world situation. In this real world study from India, we looked at the more realistic scenario of GLP-1 RA therapy being instituted when oral medicines have been suboptimal and this is the first data using this combination in this sequence.

Since it was a dual arm comparative study we could assess both the impact of adding a GLP-1 RA to a SGLT-2 inhibitor based regimen as well as compare the metabolic impact of liraglutide versus dulaglutide.

Both liraglutide as well as dulaglutide resulted in a significant reduction in fasting plasma glucose (− 41.87 ± 12.72 mg/dL with dulaglutide and − 44.20 ± 8.05 mg/dL with liraglutide), HbA1c (− 1.017 ± 0.22% with dulaglutide and − 1.547 ± 0.22%) as well as weight (− 4.20 ± 0.47 kg with dulaglutide and − 5.83 ± 0.87 kg with liraglutide) from baseline. Only liraglutide not dulaglutide had a significant impact on the reduction of systolic blood pressure (− 10.23 ± 2.36 mm of Hg). It should be noted here that all patients in this cohort had a reasonably well-controlled blood pressure at baseline and no antihypertensive medications were changed during the study.

This data would therefore suggest that as a first injectable, GLP-1 RA is a very effective option when it is used along with a SGLT-2 inhibitor on background metformin therapy in a sequential approach.

Our study also looked at the comparative effectiveness of liraglutide versus dulaglutide when added sequentially to a SGLT-2 inhibitor and metformin. There seems to be a trend favoring liraglutide in comparison to dulaglutide in reduction of fasting plasma glucose, HbA1c, weight and systolic blood pressure (statistically non significant) (Table). However, there was a statistically significant difference favoring liraglutide (63.3% with liraglutide and 30% with dulaglutide) in the proportion of patients achieving HbA1c less than 7.0%.

The modern goal of therapy is to achieve target HbA1c without gaining weight. Larger proportion of patients achieved this end point on liraglutide. But this did not reach statistical significance probably due to the small number of patients.

In all the CV outcome trials there was very modest impact on weight that could not have contributed to the CV benefits. However, the LOOK-AHEAD trial provided us some insight that a weight loss of more than 10% from baseline has the potential to alter the CV risks substantially [11]. In our study this degree of weight loss was achieved in 13.4% in the liraglutide arm.

Off note, no serious adverse events were noted during the study and the combination of metformin, SGLT2i with GLP-1 RA, seems to be efficacious and also reasonably well tolerated. Off the 60 patients who had been commenced on this therapy, no dropouts were seen, indicating that this therapy would be quite well accepted if the patients were chosen and counseled properly.

There are several limitations in this study. Firstly it was open label and not controlled. Hence this data could not correct for numerous confounding factors that could have influenced the results. Secondly the small sample size could have influenced the quantum of metabolic impact. We are aware that this size was small also because only a small percentage of our patients could afford this very expensive therapy in our “pay from pocket” setting and were “chosen” based on their ability to afford this treatment. This would also be considered a major selection bias. But in spite of these limitations this data definitely points to the safety and efficacy of using GLP1RA on a background of metformin and SGLT2i in poorly controlled diabetes and this method of treatment could be further tested in larger trials.

## Conclusion

In this retrospective study in Indian type 2 diabetic patients poorly controlled with metformin and SGLT-2 inhibitor we found a meaningful impact of adding a GLP-1 RA on all metabolic parameters. Both liraglutide and dulaglutide were effective in this regard. There were additional advantages seen with liraglutide as far achieving target HbA1c of less than 7% and also on the quantum of weight loss and systolic blood pressure reduction.

This study therefore provides a pilot and indeed generates a hypothesis supporting the sequential addition of a GLP-1 RA after metformin and SGLT-2 inhibitor, in the management of T2D, which needs to be tested in a more systematic manner on a larger population.

## Abbreviations

BMI: Body mass index
CVOT: Cardiovascular outcome trials
GLP-1RA: Glucagon like Peptide Receptor Analogues
HbA1c: Glycosylated hemoglobin
HPLC: High performance liquid chromatography
OAD: Oral antidiabetic drugs
SGLT-2i: Sodium-glucose linked transporter inhibitor
T2D: Type 2 diabetes
